# The Reduced Gut *Lachnospira* Species Is Linked to Liver Enzyme Elevation and Insulin Resistance in Pediatric Fatty Liver Disease

**DOI:** 10.3390/ijms25073640

**Published:** 2024-03-25

**Authors:** Ching-Chung Tsai, Min-Hsi Chiu, Ho-Poh Kek, Ming-Chun Yang, Yu-Tsun Su, Hsien-Kuan Liu, Ming-Shiang Wu, Yao-Tsung Yeh

**Affiliations:** 1 Department of Pediatrics, E-Da Hospital, I-Shou University, No. 1, Yi-Da Road, Yan-Chao District, Kaohsiung City 82445, Taiwan; u101130@gmail.com (C.-C.T.); k017059@gmail.com (H.-P.K.); mcyangntu@gmail.com (M.-C.Y.); suyutsun@yahoo.com.tw (Y.-T.S.); jonathan730608@yahoo.com.tw (H.-K.L.); 2 School of Medicine for International Students, College of Medicine, I-Shou University, No. 8, Yi-Da Road, Yan-Chao District, Kaohsiung City 82445, Taiwan; 3 Aging and Disease Prevention Research Center, Fooyin University, No. 151, Jinxue Road, Daliao District, Kaohsiung City 83102, Taiwan; c12010521@yahoo.com.tw; 4 Department of Medical Laboratory Science and Biotechnology, Fooyin University, No. 151, Jinxue Road, Daliao District, Kaohsiung City 83102, Taiwan; 5 School of Medicine, College of Medicine, I-Shou University, No. 8, Yi-Da Road, Yan-Chao District, Kaohsiung City 82445, Taiwan; 6 Department of Internal Medicine, National Taiwan University Hospital and National Taiwan University College of Medicine, No. 7, Zhongshan S. Road, Zhongzheng District, Taipei City 100225, Taiwan; mingshiang@ntu.edu.tw

**Keywords:** pediatric obesity, insulin resistance, microbiome diversity, inflammation, flavonoids

## Abstract

The objective of this study was to investigate gut dysbiosis and its metabolic and inflammatory implications in pediatric metabolic dysfunction-associated fatty liver disease (MAFLD). This study included 105 children and utilized anthropometric measurements, blood tests, the Ultrasound Fatty Liver Index, and fecal DNA sequencing to assess the relationship between gut microbiota and pediatric MAFLD. Notable decreases in *Lachnospira* spp., *Faecalibacterium* spp., *Oscillospira* spp., and *Akkermansia* spp. were found in the MAFLD group. *Lachnospira* spp. was particularly reduced in children with MAFLD and hepatitis compared to controls. Both MAFLD groups showed a reduction in flavone and flavonol biosynthesis sequences. *Lachnospira* spp. correlated positively with flavone and flavonol biosynthesis and negatively with insulin levels and insulin resistance. Body weight, body mass index (BMI), and total cholesterol levels were inversely correlated with flavone and flavonol biosynthesis. Reduced *Lachnospira* spp. in children with MAFLD may exacerbate insulin resistance and inflammation through reduced flavone and flavonol biosynthesis, offering potential therapeutic targets.

## 1. Introduction

Childhood obesity is a global public health issue that has become more prevalent and affects younger age groups. The condition stems from multiple factors, such as genetic predisposition, unhealthy dietary patterns, lifestyle choices, and environmental and societal influences [1,2,3]. Childhood obesity poses a substantial risk for the development of adult obesity and several severe medical conditions, such as type 2 diabetes, cardiovascular disease, and metabolic dysfunction-associated fatty liver disease (MAFLD) [4,5,6]. Furthermore, childhood obesity has been linked to the emergence of various non-metabolic health issues, such as certain types of cancers, obstructive sleep apnea, asthma, infertility, polycystic ovary syndrome, orthopedic complications, and psychiatric disorders [7,8,9,10].

MAFLD, a prevalent chronic liver disease, is estimated to affect 34.2% of obese children and adolescents aged between 1 and 19 years and 7.6% of the general pediatric population [2,11]. It is a multifactorial condition that can lead to chronic hepatic inflammation, fibrosis, and ultimately cirrhosis if untreated. MAFLD is associated with fat accumulation in the liver, insulin resistance, and oxidative stress, which are influenced by various factors, such as high-sugar diets, gut dysbiosis, and gut–liver axis dysfunction [12,13]. Recent evidence has increasingly highlighted the roles of the gut–liver axis, a complex interplay between the gut and liver that is mainly modulated by the gut microbiota, in the pathogenesis of MAFLD [14,15]. This interaction may involve dysbiosis of the gut microbiota, impairment of the intestinal barrier defense system, and the overgrowth of small intestinal bacteria. Dysbiosis of the gut microbiota can contribute to inflammation by altering trimethylamine, secondary bile acids, short-chain fatty acids (SCFAs), and ethanol production, subsequently increasing toxic bacterial products, pro-inflammatory cytokines, and endotoxin levels, all of which are involved in gut inflammation. A compromised intestinal barrier defense system facilitates the passage of microbial- or pathogen-related molecules into the portal venous system, allowing them to reach the liver. In the liver, these molecules activate recognition receptors such as Toll-like receptors in Kupffer and hepatic stellate cells, leading to chronic hepatic inflammation and fibrosis. Therefore, the gut microbiota plays a critical role in the pathogenesis of MAFLD [13,15,16,17].

Although MAFLD is most accurately diagnosed by a liver biopsy, the invasiveness of this procedure renders it unsuitable for routine screening in children. As an alternative, the Ultrasound Fatty Liver Index (US-FLI) has been developed as a noninvasive scoring system for assessing MAFLD. Research in adults has shown that the US-FLI is highly correlated with several markers of metabolic dysfunction and histological features of MAFLD, including nonalcoholic steatohepatitis (NASH). Furthermore, US-FLI values < 4 have a high negative predictive value for severe NASH, which may reduce the need for liver biopsy [18]. Moreover, noninvasive US-FLI is a useful tool for assessing MAFLD in children [19].

Understanding the relationship between gut microbiota dysbiosis and MAFLD is critical for developing innovative preventive and therapeutic strategies. This study aimed to investigate changes in the gut microbiota’s composition and their impact on the metabolism of children with MAFLD, including the underlying mechanisms. The ultimate objective of this study was to apply these findings to the development of prophylactic and remedial approaches for children with MAFLD.

## 2. Results

### 2.1. Presentation of Gut Dysbiosis in Children with MAFLD Compared to Those without MAFLD

**Figure 1:**
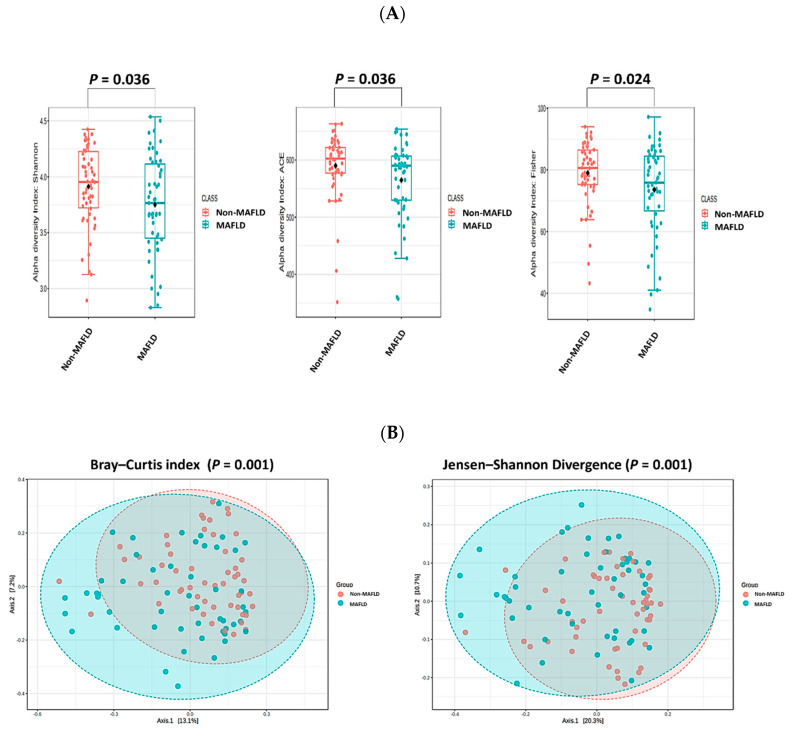
Comparison of alpha (α) and beta (β) diversity of the bacterial community between metabolic dysfunction-associated fatty liver disease (MAFLD) and non-MAFLD groups. (**A**) Alpha diversity indices (Shannon, Abundance-based Coverage Estimator (ACE), and Fisher) were significantly lower in the MAFLD group. (**B**) Significant differences in beta diversity indices (Bray–Curtis and Jensen–Shannon divergence) were observed. Analyses were performed using Microbiome Analyst. *p* < 0.05 indicates statistical significance.

**Figure 2:**
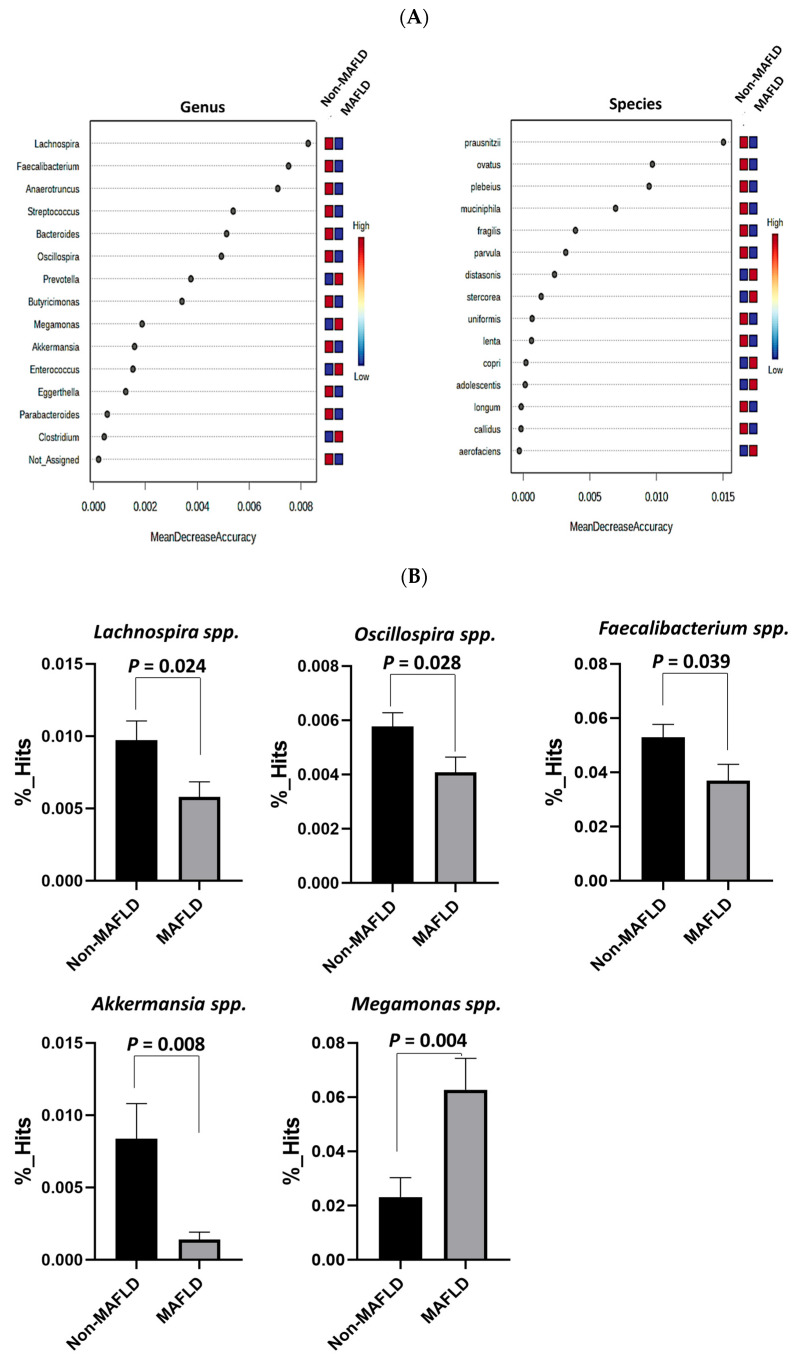

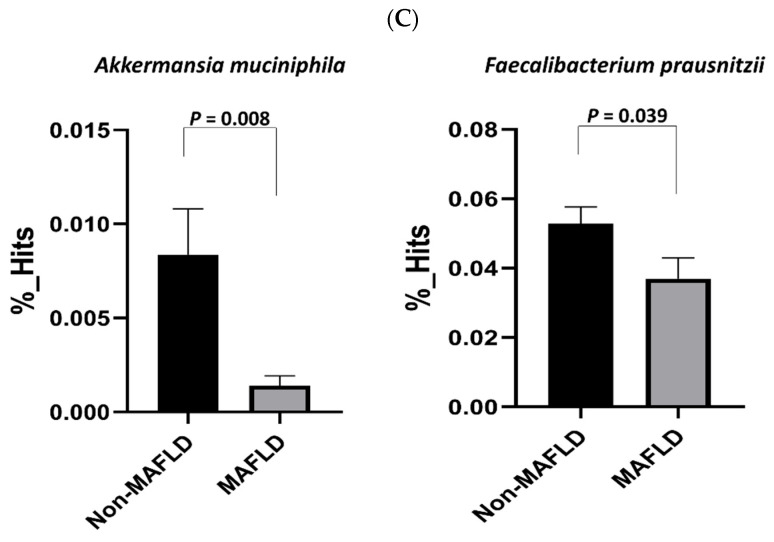
Comparison of the mean decrease accuracy and composition of gut microbiota between metabolic dysfunction-associated fatty liver disease (MAFLD) and non-MAFLD groups. (**A**) The random forest variable importance plot identifies the most important genera and species based on the mean decrease accuracy. (**B**) Genus level and (**C**) species level abundance differences are depicted. Data are presented as mean ± standard error of mean. *p* < 0.05 indicates statistical significance.

Table 1 shows that there were no significant differences in age, sex, height, or high-density lipoprotein cholesterol (HDL-C), low-density lipoprotein cholesterol (LDL-C), glycated hemoglobin (HbA1c), and fasting blood glucose levels between the MAFLD and non-MAFLD groups (*p* > 0.05). However, individuals in the MAFLD group (*n* = 51) had significantly higher values for weight, body surface area, body mass index (BMI), waist circumference, hip circumference, waist-to-hip ratio (WHR), systolic blood pressure, diastolic blood pressure, high-sensitivity C-reactive protein (hsCRP) level, triglyceride level, total cholesterol (T-CHO) level, T-CHO/HDL-C ratio, aspartate aminotransferase (AST) level, alanine aminotransferase (ALT) level, γ-glutamyltransferase (γ-GT) level, insulin level, and the modified homeostasis model assessment of insulin resistance (HOMA-IR) compared to those in the non-MAFLD group (*p* < 0.05; *n* = 54). As shown in Figure 1A, the alpha diversity, including the Shannon, Abundance-based Coverage Estimator, and Fisher indices, was significantly lower in the MAFLD group than in the non-MAFLD group. Furthermore, the beta diversity of the microbial composition, represented by the Bray–Curtis index and Jensen–Shannon divergence, showed significant differences between the MAFLD and non-MAFLD groups (Figure 1B). The results of the mean decrease in the accuracy analysis (i.e., random forest (RF) model) indicated that *Prevotella* spp. and *Megamonas* spp. at the genus level, as well as *Distasonis* and *Stercoreus* at the species level, were the most important taxa in the MAFLD group, as shown in Figure 2A. Moreover, *Lachnospira* spp., *Faecalibacterium* spp., *Oscillospira* spp., and *Akkermansia* spp. were significantly less abundant (Figure 2B, all *p* < 0.05), whereas *Prevotella* spp. and *Megamonas* spp. were significantly more abundant in the MAFLD group than in the non-MAFLD group (Figure 2B, all *p* < 0.05). As shown in Figure 2C, *Faecalibacterium prausnitzii* and *Akkermansia muciniphila* were significantly less abundant in the MAFLD group than in the non-MAFLD group (both *p* < 0.05).

### 2.2. MAFLD with Hepatitis versus MAFLD without Hepatitis

**Figure 3:**
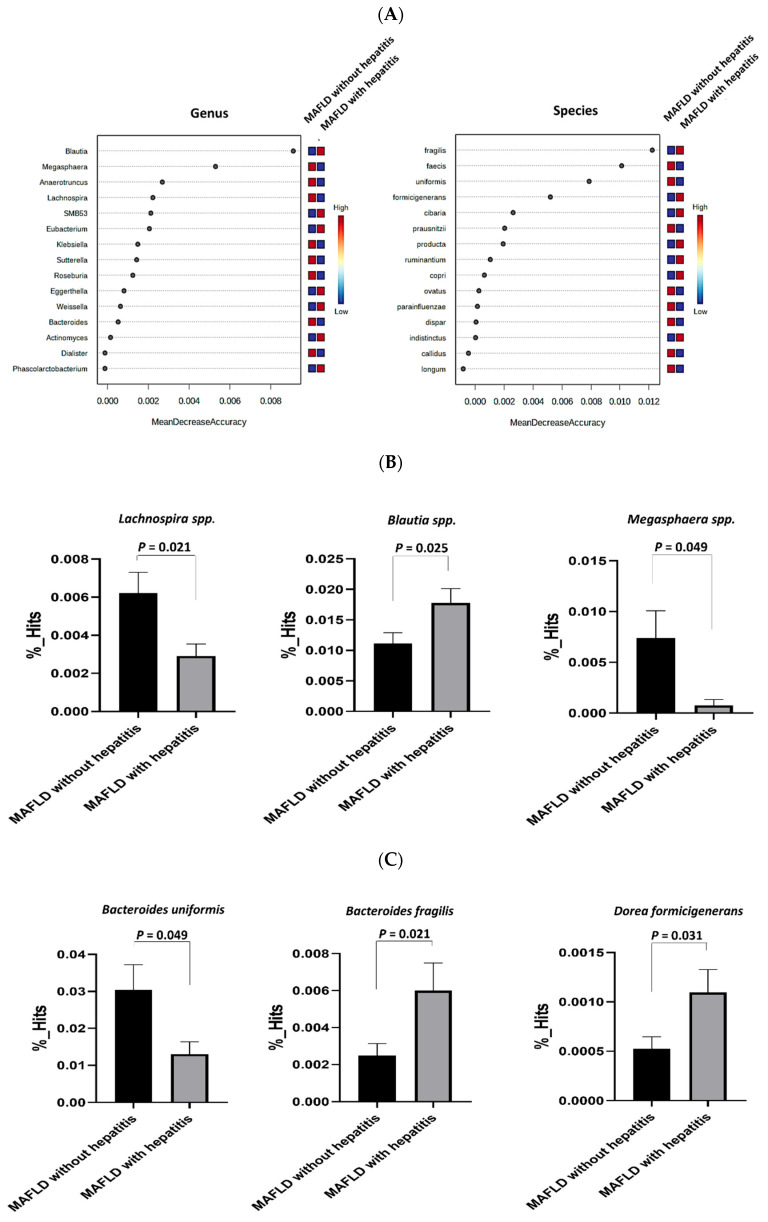
Comparison of the mean decrease accuracy and composition of gut microbiota between metabolic dysfunction-associated fatty liver disease (MAFLD) with hepatitis and MAFLD without hepatitis groups. (**A**) The random forest variable importance plot identifies the most important genera and species. (**B**) Variances at the genus level and (**C**) species level in microbiota are shown. Data are mean ± standard error of mean. *p* < 0.05 indicates statistical significance.

As shown in Figure 3A, the mean decrease accuracy analysis identified *Blautia* and *SMB53* at the genus level, as well as *Fragilis* and *Formicigenerans* at the species level, as the most important taxa in the MAFLD with hepatitis group compared to the MAFLD without hepatitis group. Figure 3B further demonstrates that *Lachnospira* spp. and *Megasphaera* spp. were significantly less abundant in the MAFLD with hepatitis group, whereas *Blautia* spp. were significantly more abundant in the same group than in the MAFLD without hepatitis group (all *p* < 0.05). Furthermore, Figure 3C shows that *Bacteroides uniformis* was significantly less abundant, and *Bacteroides fragilis* and *Dorea formicigenerans* were significantly more abundant, in the MAFLD with hepatitis group (both *p* < 0.05).

### 2.3. Predictive Functional Analysis

**Figure 4:**
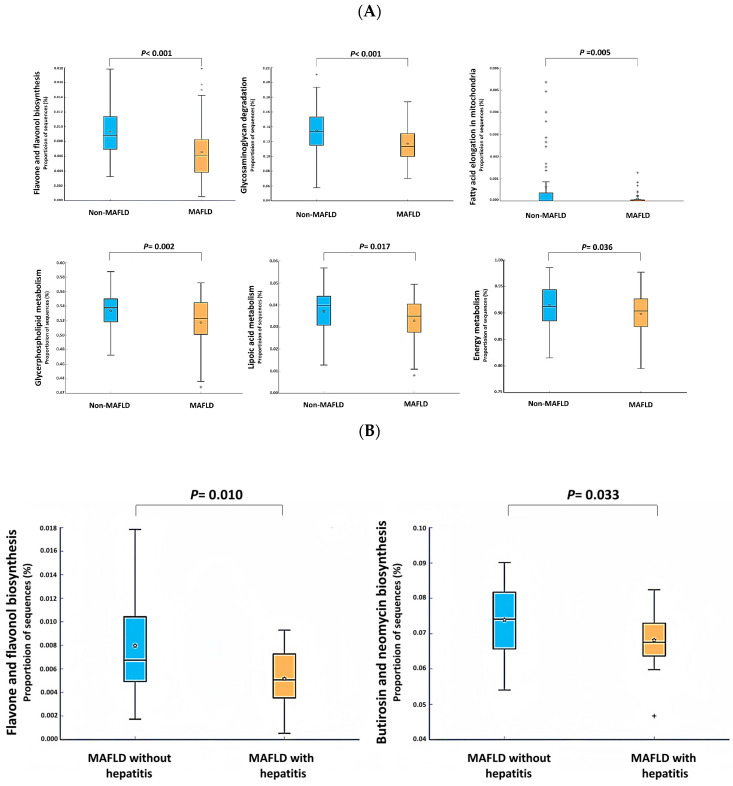

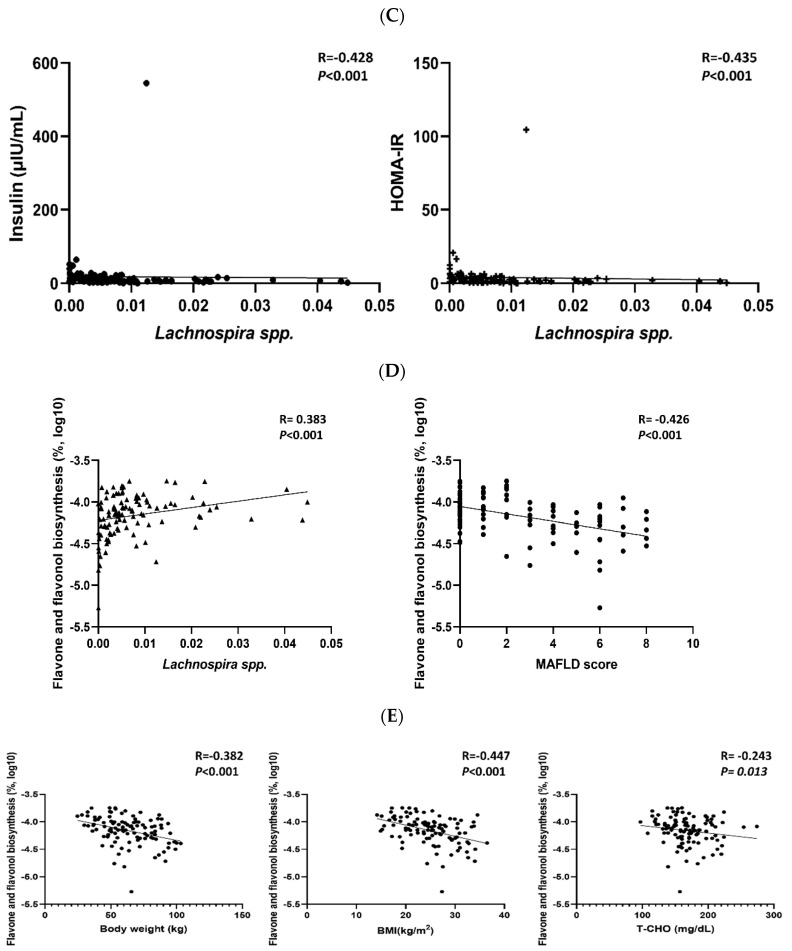
Comparison of predicted functions of gut microbiota in (**A**) metabolic dysfunction-associated fatty liver disease (MAFLD) versus non-MAFLD and (**B**) MAFLD with versus without hepatitis, analyzed using Phylogenetic Investigation of Communities by Reconstruction of Unobserved States (PICRUSt). (**C**) Correlations between *Lachnospira* spp., insulin levels, and modified homeostasis model assessment of insulin resistance (HOMA-IR). (**D**) Correlations between *Lachnospira* spp. and flavone and flavonol biosynthesis. (**E**) Correlations of the biosynthesis of flavones and flavonols with body weight, body mass index (BMI), and total cholesterol, analyzed using Spearman’s correlation. *p* < 0.05 indicates statistical significance. The symbol ‘☆’ represents the mean value, and ‘+’ denotes outliers.

As shown in Figure 4A, there was a significant reduction in the proportion of sequences associated with the biosynthesis of flavones and flavonols, energy production processes, mitochondrial fatty acid extension, glycerophospholipid metabolism, the breakdown of glycosaminoglycans, and lipoic acid metabolism in the MAFLD group compared to in the non-MAFLD group (all *p* < 0.05). Similarly, Figure 4B demonstrates a statistically significant decrease in the proportion of sequences associated with the biosynthesis of flavones and flavonols, as well as butirosin and neomycin biosynthesis, in the MAFLD with hepatitis group compared to the MAFLD without hepatitis group (all *p* < 0.05).

### 2.4. The Relationship between Gut Microbiota and Anthropometric Measurements as Well as Biochemical Data

As shown in Figure 4C, there was a significant negative correlation between *Lachnospira* spp. and insulin (R = −0.428, *p* < 0.001), as well as between *Lachnospira* spp. and HOMA-IR (R = −0.435, *p* < 0.001), while there was a negative correlation between *Lachnospira* spp. and the US-FLI score (R = −0.177, *p* = 0.072). As shown in Figure 4D, there was a significant positive correlation between *Lachnospira* spp. and flavone and flavonol biosynthesis (R = 0.383, *p* < 0.001), and a significant negative correlation between the US-FLI score and flavone and flavonol biosynthesis (R = −0.426, *p* < 0.001). Figure 4E shows a significant negative correlation between flavone and flavonol biosynthesis and weight, BMI, and T-CHO level (R = −0.382, *p* < 0.001; R = −0.447, *p* < 0.001; and R = −0.243, *p* < 0.05, respectively).

Supplemental Figure S1 shows a heatmap of Spearman correlations between metabolic parameters, gut microbiota composition, and clinical markers. Red and blue cells indicate positive and negative correlations, respectively, with the color intensity corresponding to the strength of the correlation from −1.00 to 1.00. Asterisks denote significant correlations (*p* < 0.05). The parameters include fatty acid metabolism, flavonoid biosynthesis, lipid pathways, bacterial genus abundances, inflammatory markers (hsCRP), lipid levels (TG and T-CHO), and anthropometrics (BMI). This visualization allows for an evaluation of the relationships between the host metabolism, gut microbiome, and cardiometabolic health indices.

## 3. Discussion

In the present study, the microbial alpha diversity was lower in the MAFLD than the non-MAFLD group, and the two groups showed distinct differences in microbial beta diversity. Similar results were observed in children with MAFLD compared to those with NASH or obese individuals without MAFLD [20]. As seen in other studies, the dysbiosis of gut microbiota may critically contribute to the disease’s development and progression in children [12,13,15,17].

Based on the RF model and further comparisons, *Lachnospira* spp., *Faecalibacterium* spp., *Oscillospira* spp., and *Akkermansia* spp. were less abundant, and *Prevotella* spp. and *Megamonas* spp. were more abundant in the MAFLD group. Notably, *Faecalibacterium prausnitzii* and *Akkermansia muciniphila* were significantly decreased in the MAFLD group. In addition to *Lachnospira* spp., this study identified a lower abundance of beneficial gut microbiota, including butyrate-producing *Faecalibacterium* spp. and *Faecalibacterium prausnitzii,* and gut-protective *Akkermansia* spp. and *Akkermansia muciniphila*, in individuals with MAFLD than in those without MAFLD.

Previous studies have reported that *Faecalibacterium* spp. ferments dietary fiber to produce short-chain fatty acids (SCFAs) such as butyrate, which is beneficial in protecting against MAFLD [21,22,23]. Similarly, *Faecalibacterium prausnitzii* has a protective effect against MAFLD [22,23,24]. Notably, the abundance of *Faecalibacterium* is significantly lower in children with MAFLD or NASH [25]. Therefore, the results of the present study suggest that the lower abundance of *Faecalibacterium* spp. and *Faecalibacterium prausnitzii* may be associated with the development of MAFLD in individuals, potentially owing to a reduction in butyrate production. Intriguingly, decreased levels of *Akkermansia* compared with those of healthy children were also observed in other studies [20,26]. *Akkermansia muciniphila* regulates triglyceride synthesis and maintains gut homeostasis, preventing fatty liver disease in obese mice [27]. Supplementation with *Akkermansia muciniphila* can prevent hepatic fat accumulation and inflammation in patients with MAFLD [28]. *Akkermansia muciniphila* may have a direct beneficial effect on the gut epithelium and promote butyrate production when co-cultured with other butyrate-producing bacteria such as *Faecalibacterium prausnitzii* [17]. In this study, *Akkermansia muciniphila* was positively correlated with fatty acid elongation in the mitochondria, which was negatively associated with HOMA-IR, suggesting that the decreased abundance of *Akkermansia muciniphila* might indirectly contribute to increased insulin resistance in children with MAFLD. Although these findings suggest that dysbiosis may contribute to MAFLD development and progression in children, the composition of the gut microbiota varies between populations; therefore, making conclusive or causative claims is challenging.

This study revealed that the abundance of the *Lachnospira* spp. was significantly lower in the MAFLD and MAFLD with hepatitis groups than in the corresponding control groups. Similar observations have been reported in previous studies, where a reduced abundance of *Lachnospira* spp. was noted in individuals with MAFLD compared to that in healthy controls [29,30]. Notably, our findings also showed a decreased abundance of *Lachnospira* spp. in the obese group, although the corresponding data are not presented in this study. These observations suggest a possible association between *Lachnospira* spp., individuals with obesity, and MAFLD. In light of the protective role of *Lachnospiraceae* in MAFLD, as suggested by Zhang et al. [31], our study provides a nuanced examination of how specific *Lachnospira* species interact with pediatric fatty liver disease. While Zhang et al. emphasize *Lachnospiraceae’s* general protective effects, our findings reveal a more complex scenario, where the diminished presence of particular *Lachnospira* species is associated with increased liver enzyme levels and insulin resistance in children with MAFLD. This distinction emphasizes microbial specificity in disease mechanisms and opens doors for targeted therapies. These insights could lead to precision medicine in MAFLD, aiming to modulate specific microbial communities to slow disease progression.

The results of this study reveal a significant negative correlation between the abundance of *Lachnospira* spp. and insulin resistance, as evidenced by the significant negative association between the abundance of *Lachnospira* spp. and serum insulin and HOMA-IR levels. These findings suggest that the decreased abundance of *Lachnospira* spp. may be associated with increased insulin resistance, which is a known risk factor for MAFLD development. This is consistent with previous studies [13,32].

Given our findings, understanding how a reduced abundance of *Lachnospira* spp. and beneficial gut microbiota contributes to insulin resistance and inflammation in pediatric MAFLD is crucial. Our study suggests a link between fewer *Lachnospira* spp. and a decreased biosynthesis of flavones and flavonols—compounds with anti-inflammatory and insulin-sensitizing properties [33]. This indicates a mechanism by which changes in the gut microbiota, notably a decrease in *Lachnospira* spp., could diminish flavonoid biosynthesis, potentially leading to increased inflammation and insulin resistance. Modulating the gut microbiome to replenish *Lachnospira* spp. and other beneficial bacteria might offer a new strategy for combating insulin resistance and inflammation in children with MAFLD.

The precise mechanisms underlying the effects of *Lachnospira* spp. on the development of MAFLD are not fully understood. However, butyrate production may help to protect against MAFLD development because butyrate is known to promote intestinal health and have anti-inflammatory, antioxidative, and insulin-sensitizing effects [22,23,34]. Several studies have also linked *Lachnospira* spp. to bile acid metabolism, which is essential for the absorption and metabolism of dietary lipids and is associated with MAFLD development [35,36]. In addition, this study suggests that insulin resistance is a potential mechanism underlying the association between *Lachnospira* spp. and the development of MAFLD.

Flavones and flavonols are flavonoids, a group of natural plant compounds. Research has suggested that certain flavones, such as apigenin, scutellarin, tangeretin, and acacetin, may improve MAFLD by regulating lipid metabolism, reducing oxidative stress and inflammation, and enhancing autophagy [37,38,39,40]. Similarly, luteolin, a flavone, alleviates MAFLD in rats by helping to repair intestinal mucosal barrier damage and addressing microbiota imbalances in the gut–liver axis [41,42]. Flavonols have a similar beneficial effect in MAFLD. Studies have shown that hyperoside attenuates MAFLD in rats by regulating cholesterol and bile acid metabolism and targeting Nr4A1 in macrophages [43,44]. Kaempferol and kaempferide, on the other hand, attenuate oleic-acid-induced lipid accumulation and oxidative stress in HepG2 cells [45]. Additionally, studies have shown that increased flavonoid consumption is linked to a decreased likelihood of MAFLD advancement in the elderly overweight or obese Chinese population and animal models [46,47]. Vegetarian and vegan diets may benefit patients with MAFLD due to the correlation between plant-based diets and *Lachnospira* spp., whereas omnivorous diets have a negative correlation [48]. Furthermore, previous studies have shown that vitamin D supplementation increases the abundance of *Lachnospira* spp. and decreases the abundance of *Blautia* spp. [49], which may help correct the dysbiosis observed in our study. However, further research is required to clarify the potential therapeutic effects of plant-based diets and vitamin D supplementation on MAFLD. Intriguingly, this study also found that the abundance of *Blautia* spp., an SCFA producer, was discordant in the group with MAFLD and hepatitis [16,50,51,52].

In the present study, a significant inverse relationship between flavone and flavonol biosynthesis, weight, BMI, and T-CHO levels was observed. Previous research suggests that flavonoids can potentially exert anti-obesity effects by impacting various factors, including food intake, enzyme activities, nutrient absorption, adipocyte development and function, thermogenesis, energy consumption, and intestinal microbiota composition [53]. Additionally, flavonols have the ability to lower T-CHO levels [54]. Therefore, this study suggests that the association between flavone and flavonol biosynthesis and the development of MAFLD may be linked to obesity and hypercholesterolemia.

This study demonstrated a significant positive association between the abundance of *Lachnospira* spp. and the biosynthesis of flavones and flavonols. Conversely, there was a significant negative correlation between flavone and flavonol biosynthesis and the US-FLI score, whereas there was a negative correlation between the *Lachnospira* spp. abundance and US-FLI score. These findings suggest a close relationship between *Lachnospira* spp. abundance, flavone and flavonol biosynthesis, and MAFLD development. Nevertheless, further investigation is warranted to confirm this relationship.

This study had several limitations. First, liver biopsies for fatty liver identification in children were not performed because of the difficulty in obtaining parental agreement. Second, although there was a significant decrease in the abundance of *Lachnospira* spp. and flavone and flavonol biosynthesis in both the MAFLD and MAFLD with hepatitis groups compared to that in the corresponding control group, the causative relationship between *Lachnospira* spp. and flavone and flavonol biosynthesis remains unknown. Furthermore, whether *Lachnospira* spp.; flavone, flavonol, or vitamin D supplementation; or plant-based diets improve MAFLD in children is unclear. Further studies are required to explore this relationship and its potential therapeutic effects.

This study revealed a decrease in the levels of *Lachnospira* spp. and sequences related to flavone and flavonol biosynthesis in both the MAFLD and MAFLD with hepatitis groups. *Lachnospira* spp. showed a negative correlation with insulin resistance, whereas flavones and flavonol biosynthesis-related sequences were negatively correlated with obesity and hypercholesterolemia, which are risk factors for MAFLD. However, additional research is necessary to understand the connection between *Lachnospira* spp. and flavone/flavonol biosynthesis, as well as their possible therapeutic benefits in children with MAFLD.

## 4. Materials and Methods

### 4.1. Study Design

Between July 2019 and June 2020, a prospective study was conducted at the outpatient department of E-Da Hospital with 122 volunteers aged 9–18 years. Participants with a history of hepatitis B or C; systemic disease; recent febrile illness; major surgery; recent probiotic, medication, or antibiotic use; or exposure to hepatotoxic agents were excluded. Furthermore, participants and their guardians were screened for alcohol consumption, and those with a history of alcohol use were excluded. Of the 122 participants, 17 did not provide sufficient fecal or blood samples, leaving 105 children for the study. The Institutional Review Board of E-Da Hospital approved the study (approval number: EMRP64106N) in compliance with the Declaration of Helsinki, and all the participants and their parents or guardians provided written informed consent. Prior to analysis, all participant information was de-identified.

### 4.2. Anthropometric Measurements and Biochemical Data

At enrollment, anthropometric measurements, including weight, height, waist/hip circumference, and blood pressure, were obtained from the participants after removing shoes and heavy clothing. The BMI was calculated using standard methods, and the WHR and waist-to-height ratio were determined. Venous blood samples were collected at E-Da Hospital after overnight fasting to test for hepatitis B surface antigen and anti-hepatitis C virus antibodies; perform liver function tests (i.e., AST, ALT, hsCRP, and γ-GT levels); obtain blood lipid profiles (i.e., T-CHO, triglyceride, HDL-C, and LDL-C levels); and determine HbA1c, fasting blood glucose, and insulin levels. Insulin resistance was assessed using the HOMA-IR formula: (fasting plasma glucose level [mmol/L] × fasting serum insulin level [mU/L])/22.5 [55]. Participants with positive results for the hepatitis B surface antigen and anti-hepatitis C virus antibodies were excluded.

### 4.3. Ultrasonographic Assessment for MAFLD

The scores for the US-FLI, a tool used to semiquantitatively assess the presence of fatty liver, range from 2 to 8. Diagnosing fatty liver using the US-FLI requires liver/kidney contrast for steatosis, which is scored as mild/moderate (2 points) or severe (3 points). Other scoring items included ultrasound beam attenuation, the blurring of blood vessel margins, identification of the gallbladder wall and diaphragm, and the detection of localized fat-spare areas. Each scoring item was assigned a value of 1 for yes and 0 for no. Therefore, the minimum score required for diagnosing MAFLD using the US-FLI was 2 [18,19]. In addition, MAFLD with hepatitis was defined as US-FLI values ≥ 2 and ALT levels ≥ 41, while MAFLD without hepatitis was defined as US-FLI values ≥ 2 and ALT levels < 41.

### 4.4. Fecal DNA Extraction

DNA from bacterial cells in stool samples was isolated through an adaptation of the QIAmp Fast DNA Stool Mini Kit (Qiagen, Hilden, Germany) method. Initially, fecal specimens were centrifuged at 13,200 rpm for 5 min to discard the storage solution, followed by cell lysis with InhibitEX buffer, proteinase K, and ethanol to procure a clear supernatant. This liquid was then purified using a QIAamp spin column and collected through elution. The DNA concentration was assessed using a NanoDrop 2000 spectrophotometer, (Thermo Fisher Scientific, Waltham, MA, USA), with subsequent tenfold dilution in elution buffer for analysis.

### 4.5. 16S rRNA Gene and Next-Generation Sequencing (NGS) Analysis

To construct the metagenomic sequencing library, the V3-V4 region of the 16S rRNA gene was targeted. This segment was amplified utilizing the KAPA HiFi HotStart ReadyMix (Roche, Basel, Switzerland), with subsequent purification of the PCR product using AMPure XP magnetic beads (Beckman Coulter, Brea, CA, USA). The integrity and size distribution of the PCR amplicons were verified using the Advanced Analytical Fragment Analyzer (Agilent Technologies, Santa Clara, CA, USA), while their concentrations were precisely measured with the Qubit 3.0 Fluorometer (Thermo Fisher Scientific, Waltham, MA, USA). Sequencing was carried out on an Illumina MiSeq platform (Illumina, San Diego, CA, USA), generating paired-end reads of 2 × 300 nucleotides and securing over 100,000 raw reads per sample.

### 4.6. Bioinformatics Analysis and Statistics

Microbiome data were analyzed by subjecting the raw paired-end reads to quality trimming and filtering using the CLC Genomic Workbench 10 (Qiagen, Germany), retaining only reads with a minimum similarity of 97% to the GreenGenes Database (v13.8). The generated operational taxonomic units (OTUs) were used to conduct taxonomic (i.e., relative abundance), alpha diversity, beta diversity (PCoA), and heatmap analyses using MicrobiomeAnalyst (https://www.microbiomeanalyst.ca/MicrobiomeAnalyst/upload/OtuUploadView.xhtml, accessed on 12 December 2023), and GraphPad Prism 8 (GraphPad Software, San Diego, CA, USA). An abundance analysis was performed using Linear Discriminant Analysis Effect Size (LEfSe), and a functional analysis was conducted using Phylogenetic Investigation of Communities by Reconstruction of Unobserved States (PICRUSt) via the Galaxy/HutLab website (http://huttenhower.sph.harvard.edu/galaxy/), accessed on 12 December 2023. Statistical significance was set at *p* < 0.05.

### 4.7. Statistical Analysis

Statistical evaluations and confirmations were conducted with IBM SPSS software (version 21; Armonk, NY, USA). Continuous data are presented in the form of mean ± standard deviation, and categorical data are depicted in number format. For continuous data assessment, either the Mann–Whitney U test or Student’s *t*-test was applied, while categorical data analyses utilized Fisher’s exact test. All the statistical analyses were based on a two-tailed test approach, with significance assigned to *p* values < 0.05.

## Figures and Tables

**Table 1:** Demographic, anthropometric, and biochemical measurements in MAFLD and non-MAFLD groups. All values are reported as mean ± standard deviation unless otherwise noted.

Variables	Non-MAFLD	MAFLD	
Sample Size	N = 54	N = 51	*p* Value
Factors associated with MAFLD in children based on US-FLI score			
Age (years)	13.19 ± 0.32	13.29 ± 0.33	0.910
Male	39 (72.2%)	41 (80.4%)	0.330
Female	15 (27.8%)	10 (19.6%)	
Body weight (kg)	57.06 ± 2.57	69.97 ± 2.38	<0.001
Body height (cm)	155.56 ± 1.84	158.64 ± 1.76	0.550
BSA (m^2^)	1.56 ± 0.04	1.75 ± 0.04	0.010
BMI (kg/m^2^)	22.77 ± 0.72	27.44 ± 0.56	<0.001
Waist circumference (cm)	78.87 ± 1.63	91.41 ± 1.58	<0.001
Hip circumference (cm)	90.73 ± 1.66	99.27 ± 1.34	<0.001
Waist/hip ratio	0.87 ± 0.01	0.92 ± 0.01	<0.001
Systolic BP (mmHg)	105.42 ± 1.74	113.22 ± 2.19	0.023
Diastolic BP (mmHg)	64.19 ± 1.25	71.82 ± 1.69	<0.001
Biochemical markers associated with MAFLD in children based on US-FLI score			
hsCRP (mg/L)	1.17 ± 0.26	2.69 ± 0.43	<0.001
Triglycerides (mg/dL)	63.13 ± 4.35	110.27 ± 12.29	<0.001
Total cholesterol (mg/dL)	160.21 ± 3.81	176.27 ± 4.59	0.006
HDL-C (mg/dL)	53.21 ± 1.57	50.55 ± 2.10	0.052
LDL-C (mg/dL)	96.56 ± 8.67	104.78 ± 4.17	0.002
TC/HDL	3.11 ± 0.10	3.70 ± 0.15	<0.001
HbA1c (%)	5.45 ± 0.04	5.58 ± 0.06	0.161
AST (U/L)	22.60 ± 0.96	40.49 ± 6.45	0.001
ALT (U/L)	18.33 ± 1.87	65.14 ± 12.72	<0.001
γ-GT (U/L)	17.33 ± 1.70	34.73 ± 3.92	<0.001
Insulin (μIU/mL)	7.79 ± 0.80	28.24 ± 10.47	<0.001
Glucose (AC) (mg/dL)	90.85 ± 0.83	93.12 ± 2.66	0.053
HOMA-IR	1.76 ± 0.18	6.33 ± 2.04	<0.001

Abbreviations: BSA, body surface area; BMI, body mass index; Bp, blood pressure; hsCRP, high-sensitivity C-reactive protein; HDL-C, high-density lipoprotein cholesterol; LDL-C, low-density lipoprotein cholesterol; TC/HDL, total cholesterol/HDL cholesterol ratio; HbA1c, glycated hemoglobin; AST, aspartate aminotransferase; ALT, alanine aminotransferase; γ-GT, gamma-glutamyl transferase; Glucose(AC), fasting blood glucose; HOMA-IR, homeostasis model assessment of insulin resistance. A *p* value less than 0.05 indicates statistical significance.

## Data Availability

All the datasets generated for this study are included in the article.

## Supplementary Materials

The following supporting information can be downloaded at: https://www.mdpi.com/article/10.3390/ijms25073640/s1.
