# Supplementary material for: The Reduced Gut Lachnospira Species Is Linked to Liver Enzyme Elevation and Insulin Resistance in Pediatric Fatty Liver Disease
Source: Int J Mol Sci. 2024 Mar 25;25(7):3640. doi: 10.3390/ijms25073640 (PMC11011648; doi:10.3390/ijms25073640)
Supplement: Supplementary file 1 [file ijms-25-03640-s001.zip › ijms-2900174-supplementary.pdf]

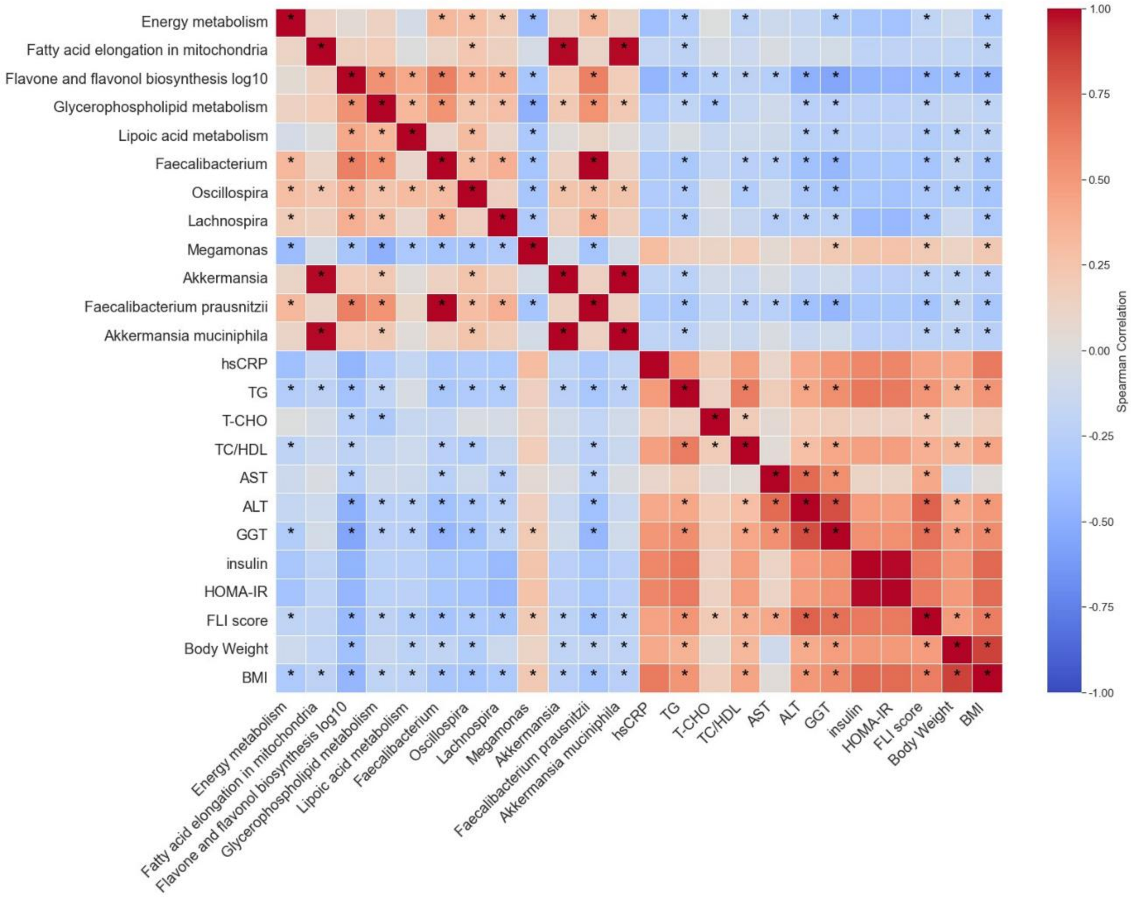

Supplemental Figure S1. This heatmap represents the Spearman coefficients of the correlation between various metabolic parameters and gut microbiota composition. Each cell shows the correlation between the parameters on the X- and Y-axes. Red indicates a positive correlation, while blue indicates a negative correlation. The intensity of the color corresponds to the strength of the correlation, with a scale from -1.00 to 1.00, as indicated by the color bar on the right. Asterisks denote statistical significance, $P<0.05$. Parameters include fatty acid elongation in mitochondria, flavone and flavonol biosynthesis, various lipid metabolisms, and the presence of specific gut microbiota genera. Clinical markers such as high-sensitivity C-reactive protein (hsCRP), triglycerides (TG), total cholesterol (T-CHO), and body composition measurements like body mass index (BMI) are also

included.
